# Up-Regulation of *MiRNA-125a-5p* Inhibits Cell Proliferation and Increases *EGFR-TKI* Induced Apoptosis in Lung Cancer Cells

**DOI:** 10.31557/APJCP.2019.20.11.3361

**Published:** 2019

**Authors:** Jamal Jamal, Neda Molaee, Hadi Karami

**Affiliations:** 1 *Molecular and Medicine Research Center, *; 2 *Traditional and Complementary Medicine Research Center, *; 3 *Department of Molecular Medicine and Biotechnology, Faculty of Medicine, Arak University of Medical Sciences, Arak, Iran. *

**Keywords:** Apoptosis, EGFR, Erlotinib, lung cancer, MiRNA, 125a-5p

## Abstract

**Background:**

Despite the dramatic efficacy of erlotinib, an *EGFR* tyrosine kinase inhibitor (TKI), most of non-small cell lung cancer (NSCLC) patients ultimately acquire resistance to this agent. Different studies indicated that *miRNA-125a-5p *is down-regulated in human lung cancer cells and may function as a tumor suppressor by targeting *EGFR*. However, the biological function of *miRNA-125a-5p* in NSCLC resistance to *EGFR-TKIs* is not fully understood. In this study the effect of *miRNA-125a-5p* on cell proliferation, apoptosis and sensitivity of the A549 lung cancer cells to erlotinib was investigated.

**Methods:**

After *miRNA-125a-5p* transfection, the expression levels of *EGFR mRNA *were measured by QRT-PCR. Trypan blue assays were performed to evaluate the proliferation of the A549 lung cancer cells. The cytotoxic effects of* miRNA-125a-5p* and erlotinib, alone and in combination, were determined using MTT assay. Combination index study was performed using the method of Chou-Talalay. Apoptosis was assessed using an ELISA cell death assay kit.

**Results:**

*MiRNA-125a-5p* clearly reduced the expression of *EGFR mRNA *in a time dependent manner, causing marked cell proliferation inhibition and spontaneous apoptosis (p<0.05, relative to control). Pretreatment with *miRNA-125a-5p* synergistically increased the cytotoxic effect of erlotinib and decreased its IC_50_. Furthermore,* miRNA-125a-5p* significantly enhanced the apoptotic effect of erlotinib. Negative control miRNA had no significant effect on biological parameter of the tumor cells.

**Conclusions:**

Our data suggest that suppression of *EGFR* by* miRNA-125a-5p* can effectively trigger apoptosis and overcome *EGFR-TKs* resistance of lung cancer cells. Therefore, *miRNA-125a-5p* may be a potential therapeutic adjuvant in patients with lung cancer.

## Introduction

Lung cancer is one of the most common cancers in terms of both incidence and mortality worldwide in men and women (Ma et al., 2016; Ashour Badawy et al., 2018). It is classified into two different groups: small cell lung cancer (SCLC), which account for the 20% of cases, and non-small cell lung cancers (NSCLC), which account for 80% (Garinet et al., 2018; Wu et al., 2019). Intrinsic resistance presents a significant challenge in the treatment of NSCLC and contributes to tumor recurrence and progression (Leonetti et al., 2019; Terlizzi et al., 2019). The epidermal growth factor receptor (*EGFR*), a member of the ErbB family of receptor tyrosine kinase (RTK), is frequently overexpressed in NSCLC and negatively correlated with poor prognosis (Barr Kumarakulasinghe et al., 2015; Hsu et al., 2019). *EGFR* signaling triggers intracellular signaling pathways such as the STAT signaling pathway, phosphoinositide 3-kinase (PI3K)/Akt pathway, and the Ras/Raf/MEK/ERK1/2 pathway, which enhances tumor cell proliferation, angiogenesis, invasion, metastasis, and apoptosis resistance (Seshacharyulu et al., 2012; Wang et al., 2013; Barr Kumarakulasinghe et al., 2015; Oronsky et al., 2018; Yang and Tam, 2018). Consequently, the* EGF*R has emerged as the target of effective cancer therapies.

*EGFR* tyrosine kinase inhibitors (TKIs), such as gefitinib and erlotinib, developed as therapeutic agents for NSCLC treatment. Despite the therapeutic benefit of *EGFR-TKIs*, all patients eventually develop resistance to these agents (Antonicelli et al., 2013; Barr Kumarakulasinghe et al., 2015; Ralki et al., 2019; Xia et al., 2019). The poor clinical response of NSCLC to anti-EGFR therapies is due to the primary and secondary resistance of cancer cells to these drugs, which is thought to occur via several mechanisms, including HER-2 amplification, MET amplification, mutation in exon 20 of *EGFR (T790M), PI3K* mutations, and transformation into small cell lung cancer (Antonicelli et al., 2013; Barr Kumarakulasinghe et al., 2015). However, the additional EGFR-TKIs resistance mechanisms had remained unclear.

*MicroRNAs (miRNAs)* are abundant class of non-coding 18-25 nucleotide small RNAs, which bind to the 3’-UTR of specific target mRNAs to suppress gene expression, either via inducing translational inhibition or mRNA degradation (Ricciuti et al., 2014; Abu-Duhier et al., 2018; Amri et al., 2019). *MiRNAs* participate in a variety of biological processes, such as cell cycle progression, proliferation, growth and apoptosis (Zhang et al., 2014; Fatima et al., 2019; Miroshnichenko and Patutina, 2019). Aberrant expression of particular *miRNAs* is a hallmark of many human tumor types, including NSCLC, and they can act as either oncogenes or as tumor suppressors (Zhao et al., 2013; MacDonagh et al., 2015; Yin et al., 2017; Bharali et al., 2018). For example, *miRNA-143* expression is strongly down-regulated in lung cancer cells, causing elevated* c-MYC, NUDT1, OCT4* and *EGFR* expression, increased tumor cell growth, metastasis and migration (Ricciuti et al., 2014; Zhang et al., 2014). In contrast,* miRNA-21* is overexpressed in different forms of cancers, including NSCLC, leading to suppression of the PTEN, increased cell growth and invasion (Wang et al., 2014; Zhang et al., 2014). Thus, miRNAs can be served as potentially useful biomarkers for the diagnosis, prognosis and treatment of lung cancer (Markou et al., 2013; Ricciuti et al., 2014; Zhang et al., 2014).

*MiRNA-125a-5p* was known as tumor suppressor that inhibits the expression of EGFR and downstream genes involved in EGFR signaling pathway, leading to inhibition of invasion and migration of lung cancer cells. Moreover, down-regulation of *miRNA-125a-5p* has been observed in several types of cancers, including lung cancer (Wang et al., 2009b; Jiang et al., 2010b; Nishida et al., 2011; Zhang et al., 2014; Wang et al., 2015). In this study, we examined the effect of *miRNA-125a-5p* on *EGFR* expression, cell proliferation and apoptosis in NSCLC cells. We hypothesized that *miRNA-125a-5p* would enhance the sensitivity of the NSCLC cells to *EGFR-TKIs *by *EGFR* silencing, and evaluated the combination effect of *miRNA-125a-5p* and erlotinib on A549 cells.

## Materials and Methods

*Cell culture*

Human lung cancer cell line A549 (Pasteur Institute, Tehran, Iran) was maintained in RPMI-1640 medium (Sigma-Aldrich, St. Louis, MO, USA) that was supplemented with 10% heat-inactivated fetal bovine serum (FBS) (Gibco; Invitrogen; Life Technologies, Germany), 1% antibiotics (100 IU/ml penicillin, 100 µg/ml streptomycin) (Sigma-Aldrich), 1% sodium pyruvate and 2 mM of glutamine at 37^o^C and 5% CO_2_.

*MiRNA transfection*

The miRNA-125a-5p mimics and negative control (NC) miRNA were ordered from Dharmacon (Lafayette, CO, USA). The sequences of miRNAs are as follows: NC miRNA: 5’-UUCUUCGAACGUGUCACGUTT-3’, *miRNA-125a*-5p: 5’-UCCCUGAGACCCUUUAACCUGUGA-3’. Just before transfection, A549 cells were cultured in RPMI-1640 medium without FBS and antibiotics. Transfection of miRNAs was performed using Lipofectamine™2000 (Invitrogen, Carlsbad, CA, USA) following the manufacturer’s instructions. Briefly, miRNAs (at a final concentration of 50 nM) and lipofectamine (4 µl/ml of transfection medium) were diluted in Opti-MEM I Reduced Serum Medium (Invitrogen) separately and incubated for 5 min at ambient temperature. Then the diluted miRNAs were mixed with the diluted Lipofectamine and incubated for another 20 min. Following on, the mixtures were added to each well containing cells and medium. After 6 h incubation of the cell culture plates at 37^o^C in CO_2_ incubator, complete growth medium was added to a final FBS concentration of 10%, with cells being incubated under the same conditions. After 48 and 48 h, down-regulation of EGFR was assessed by real-time quantitative PCR (qRT-PCR).

*QRT-PCR*

At 24, 48 and 72 h after transfection, total RNA was isolated from cells by RNA extraction kit (Takara Bio Inc., Kusatsu, Shiga, Japan) according to the manufacturer’s protocol. Complementary DNA (cDNA) was synthesized from 1 µg of total cellular RNA by use of PrimeScript 1st strand cDNA Synthesis Kit (Takara Bio Inc.) and oligo-dT primer according to the manufacturer’s recommendations. Real-time PCR was performed using SYBR Green qPCR MasterMix (Yekta Tajhiz Azma, Tehran, Iran) and a LightCycler 96 System (Roche Diagnostics GmbH, Mannhein, Germany). Each PCR reaction had the following components: 1 µl of RT product, 500 nM each of the forward and reverse primers and 10 µl of SYBR Green qPCR MasterMix. The sequences of primers used for quantitative PCR were as follows: forward, 5’-TTTACAGGAAATCCTGCATGG -3’ ,reverse, 5’- TCACTGCTGACTATGTCCC -3’, for EGFR, and forward, 5’- CTACAATGAGCTGCGTGTG -3’, and reveres, 5’- GTCTCAAACATGATCTGGGTC -3’, for β-actin. The protocol parameters were 95 °C 10 min; 95 °C 10 sec, 57°C 20 sec, 72°C 20 sec, 40 cycles. The relative transcript abundance (the amount of EGFR normalized to the β-actin) was measured using the 2^- (∆∆Ct)^ method (Livak and Schmittgen, 2001).

*Cytotoxicity assay*

The effect of miRNA-125a-5p on the sensitivity of A549 cell line to erlotinib (Sigma- Aldrich) was evaluated using 3-(4, 5-Dimethylthiazol-2-yl)-2, 5-Diphenyltetrazolium Bromide (MTT) assay. The experiment was divided into eight groups: erlotinib, *miRNA-125a-5p* mimics, *NC miRNA*, *miRNA-125a-5p *mimics and erlotinib, NC miRNA and erlotinib, miRNA blank control, erlotinib blank control and combination blank control. Briefly, cells were seeded at a density of 5×10^3^ cells/well in 96-well culture plates, and then transfected with miRNAs. Six hours after transfection, the cells were exposed to various concentrations of erlotinib (0, 2, 4, 8, 16, 32 and 64 µM). After 24 and 48 h of transfection, the cell cytotoxicity was determined using a cell MTT kit (Roche Diagnostics GmbH, Mannheim, Germany) according to the manufacturer’s protocol. The absorbance (A) of the formazan dye was measured on a microplate reader (Awareness Technology, Palm City, FL, USA) at a wavelength 570 nm. The survival rate (SR) was calculated according to the equation as follows: SR (%) = (A Test /A Control) ×100%. IC_50_ (concentration that produced 50% cytotoxicity) values of the treatments alone or in combination were determined using Prism 6.01 software (GraphPad Software Inc., San Diego, CA, USA). 

*Drug combination study*

The combination index (CI) analysis based upon the Chou-Talalay method was used to determine the interaction between *miRNA-125a-5p* and erlotinib (Chou and Talalay, 1984). The data obtained with the MTT assay was converted to Fraction affected (Fa; range 0-1; where Fa = 0 represents 100% cell survival and Fa = 1 represents 0% cell survival) and analyzed with the CompuSyn version 1.0 software (ComboSyn Inc., Paramus, NJ, USA). A CI of < 1, =1 or >1 indicates synergistic, additive and antagonistic effects, respectively.

*Cell viability assay*

The effect of miRNA-125a-5p on cell proliferation was measured by the trypan blue exclusion assay. A549 cells (5×10^ 4^ cells/well) were treated with *miRNA-125a-5p* in 24-well cell culture plates and incubated for 5 days. At indicated time points, the cells were harvested and equal volumes of cell suspension and 0.4% trypan blue solution (Merck KGaA, Darmstadt, Germany) were mixed gently. Then, the numbers of viable cells (unstained) were counted microscopically (Nikon Instrument Inc., Melville, NY, USA) using a hemocytometer. The cell viability was expressed as a percentage.

*Apoptosis ELISA assay*

The A549 cells were seeded at a density of 1×10^5^ cells/well in 12-well plates and then exposed to *miRNA-125a-5p* mimics or NC miRNA, erlotinib (IC_50_ doses of 24 and 48 h) and their combination, as described previously. At 24 and 48 h after transfection, cells were collected and apoptosis was assessed using an ELISA cell death detection kit (Roche Diagnostics GmbH) according to the manufacturer’s protocol. This assay measures the amount of nucleosomal formation produced during apoptosis. Briefly, the cell lysates were transferred into the streptlized-coated plate and incubated with a mixture of anti-DNA-peroxidase and anti-histone-biotin. Following color development with 2, 2-azino-bis (3-ethylbenzthiazoline-6-sulfonic acid) solution, the absorbances of the samples was measured with an ELISA plate reader at 405 nm (reference wavelength 540 nm). Results were expressed as the fold increase in apoptosis as compared with control group.

*Statistical analysis*

Quantitative data were presented as mean ± standard deviation (SD). Analysis of variance (ANOVA) followed by Bonferroni’s test were used to determine statistical differences between groups. Value of p less than 0.05 was considered statistically significant. All statistical analyses were performed using GraphPad Prism software.

## Results

*MiRNA-125a-5p suppressed EGFR mRNA levels in A549 cells*

**Figure 1:**
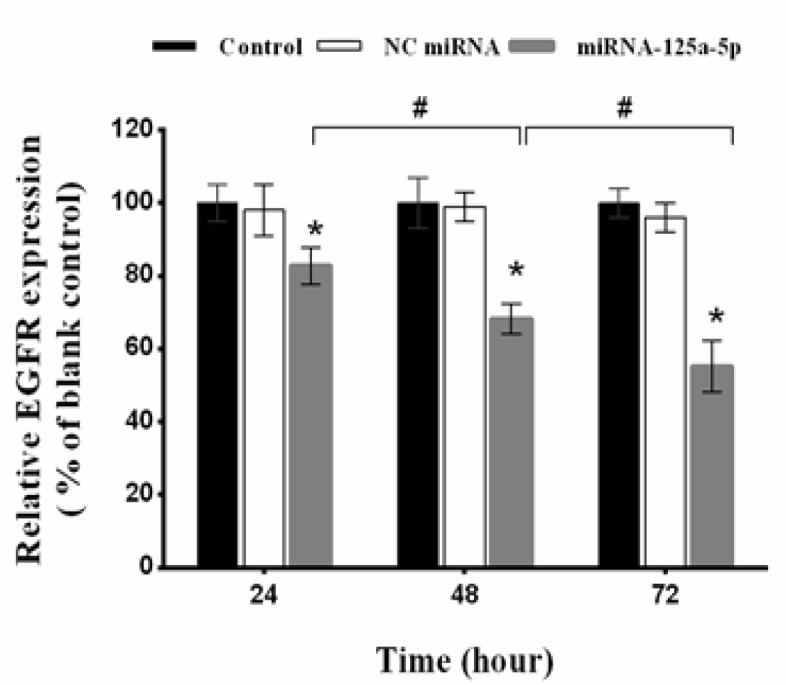
Effect of miRNA-125a-5p on the Expression of EGFR in A549 Cells. The EGFR expression determined by RT-qPCR at 24, 48 and 72 h after transfection of the cells with miRNA-125a-5p and negative control (NC) miRNA. Relative EGFR mRNA expression was measured using the 2^- (∆∆Ct)^ method. The EGFR mRNA decreased clearly at the three time points compared with corresponding blank control and NC miRNA groups (*p<0.05). The results presented are mean±SD of three independent experiments. #*p <0.05*

Firstly, we explored the effect of *miRNA-125a-5p* on *EGFR mRNA* expression in cancer cells by RT-qPCR. Relative *EGFR mRNA* expression was calculated in relation to the blank control (set at 100%). Compared with the blank control group, the expression of EGFR mRNA in A549 cells transfected with *miRNA-125a-5p *was significantly down-regulated (p<0.05; Figure 1). *MiRNA-125a-5p *reduced the *EGFR mRNA* level by 82.78%, 68.21% and 55.19% after 24, 48 and 72 h, respectively (p<0.05). Meanwhile, treatment with negative control miRNA had minimal effect on mRNA levels compared with the blank control group (p>0.05; Figure 1).

*MiRNA-125a-5p enhanced the cytotoxic effect of erlotinib in lung cancer cells*

**Figure 2:**
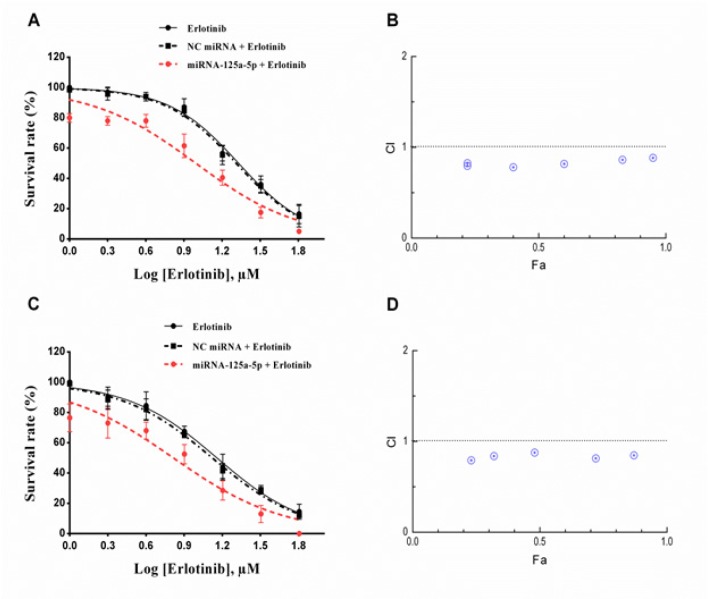
Effect of miRNA-125a-5p in Combination with Erlotinib on Cell Survival. Human A549 cells were treated with miRNA-125a-5p (50 nM) and different concentrations of erlotinib for 24 h (A and B) and 48 h (C and D). Cell survival was determined by the MTT assay as described in the method section. Dose-response curves were plotted using GraphPad Prism 6.01 software. Bars represent mean±SD (n=3). Data from three independent experiments were used to calculate the combination index (CI) according the method of Chou-Talalay. A horizontal dashed marks CI=1

To analyze whether down-regulation of *EGFR* could enhance the sensitivity of the A549 cells to erlotinib, a combination treatment of erlotinib and *miRNA-125a* was investigated. The results of MTT assay showed that monotreatment with erlotinib induced cell toxicity in a dose-dependent manner. As shown in Figure 2A and 2C, after 24 and 48 h of incubation, *miRNA-125a-5p* significantly lowered the cell survival rate to 80.30% and 76.51% respectively, relative to the blank control group (p<0.05). Moreover, erlotinib in combination with *miRNA-125a-5p* further reduced the cell survival rate relative to erlotinib or* miRNA-125a-5p* alone (p<0.05). Surprisingly, the presence of miRNA-125a-5p caused a clear reduction in the IC_50_ values of erlotinib from 21.42 µM to 9.87 µM and 14.41 µM to 6.54 µM after 24 and 48 h, respectively (Table 1). Meanwhile, transfection with NC miRNA had an insignificant effect on the sensitivity of the tumor cells relative to the erlotinib treated cells (p>0.05; Figure 2 and Table 1).

*MiRNA-125a-5p acts synergistically with erlotinib to decrease the cell survival of A549 cells*

To further examine whether the decrease in cell survival was the synergistic effect of the *miRNA-125a-5p *and erlotinib, combination index analysis was performed on MTT assay data using non-constant method of Chou and Talalay. The CI–Fa curves demonstrated a synergism (CI<1) in A549 cells when *miRNA-125a-5p* (50 nM) combined with erlotinib (2-64 µM) (Figure 2B and 2D). Our data showed that the best mean CI value of 24 h of treatment (CI=0.78) was obtained at 8 µM erlotinib with Fa level of 0.40 (Figure 2B). Moreover, at 2 µM erlotinib with Fa level of 0.23 the best mean CI value for 48 h (CI=0.79) was observed (Figure 2D).

*Up-regulation of miRNA-125a-5p inhibited cell proliferation*

**Figure 3:**
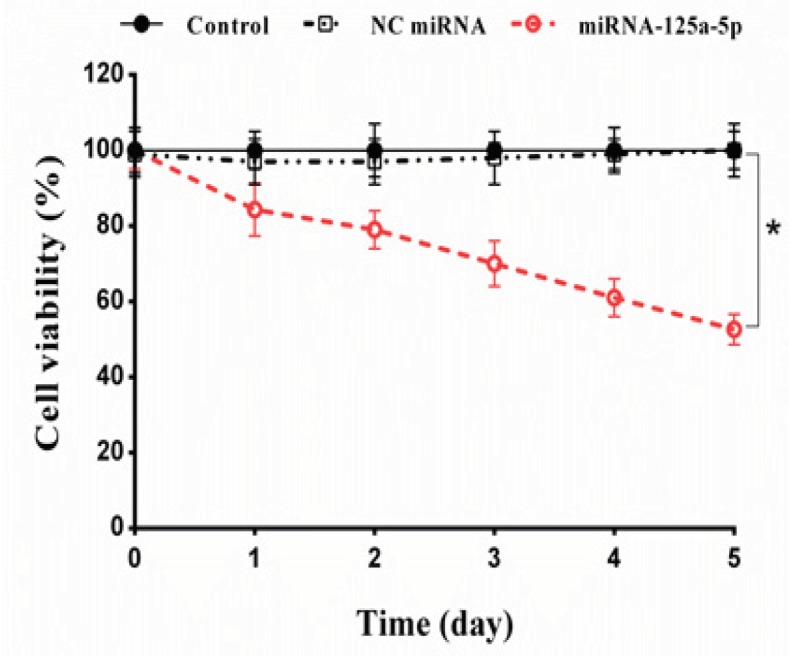
Effect of Down-Regulation of EGFR by miRNA-125a-5p on Lung Cancer Cell Proliferation. The A549 cells were transfected with miRNA-125a-5p and negative control (NC) miRNA and then cell viability was tested by trypan blue assay over a period of 5 days. The results represent mean±SD of three independent experiments. *p<0.05 versus blank control or NC miRNA

As down-regulation of *miRNA-125a-5p* is associated with survival of lung cancer cells; we therefore sought to test whether up-regulation of this miRNA could inhibit the proliferation of A549 cells. The tumor cells were transfected with *miRNA-125a-5p *and NC miRNA. Then, the cell viability was measured every 24 h for 5 days by trypan blue exclusion assay. The cell proliferation curve showed that *miRNA-125a-5p* significantly reduced cell viability compared with blank control group in a time dependent way (p<0.05; Figure 3). Twenty-four hours after transfection of *miRNA-125a-5p*, the cell viability decreased to 84.39% and dropped to 52.61% on day 5. In contrast, no significant alterations in cell proliferation were detected between the NC miRNA and the blank control groups (p>0.05; Figure 3).

*MiRNA-125a-5p sensitized lung cancer cells to apoptosis induced by erlotinib*

**Figure 4:**
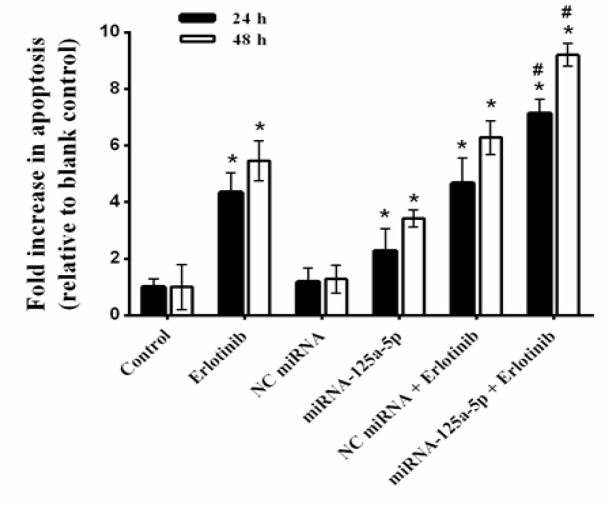
Cell Apoptosis of A549 Cells Treated with miRNA-125a-5p and Erlotinib. The cells were treated with miRNA-125a-5p (50 nM), negative control (NC) miRNA (50 nM) and erlotinib (IC_50_ doses of 24 and 48 h), alone and in combination, and then apoptosis was measured by cell death ELISA. The data are expressed as mean±SD (n=3); *p<0.05 versus blank control or NC miRNA; #*p<0.05 *versus miRNA-125a-5p or erlotinib

To confirmed investigate whether the sensitizing effect of the *miRNA-125a-5p* was related to the increase in the extent of apoptosis, the effects of* miRNA-125a-5p*, erlotinib and their combination on apoptosis, were evaluated using an ELISA-based cell death detection system. As shown in Figure 4, 24 h after transfection of *miRNA-125a-5p* alone, apoptosis enhanced by 2.27 fold, whereas erlotinib treatment alone caused 4.35 fold increase in apoptosis (p<0.05, compared to the blank control). In contrast, the combination treatment further enhanced apoptosis to 7.14 fold (p<0.05, compared with single agent treatment). Moreover, after 48 h of treatment of A549 cells to* miRNA-125a-5p *or erlotinib alone, apoptosis increased by 3.43 and 5.46 fold, respectively, compared to the blank control (p<0.05). Also, combination therapy further enhanced apoptosis to 9.21 fold after 48 h (Figure 4; p<0.05, relative to the blank control or monotreatment). On the other hand, treatment with *NC miRNA* alone or in combination with erlotinib displayed no significant alterations in the extents of apoptosis compared with the blank control or erlotinib monotreatment, respectively. Therefore, these results indicate that the sensitization effect of *miRNA-125a-5p* is partially attributed to the induction of apoptosis.

**Table 1:** Half Maximal Inhibitory Concentration (IC_50_) of Erlotinib Alone and in Combination with miRNAs, after 24 and 48 h of Treatment

Treatment	IC_50_	
	24 h	48 h
Erlotinib	21.42 ± 1.10	14.41 ± 2.10
NC miRNA and erlotinib	20.49 ± 1.17#	13.25 ± 1.30#
miRNA-125a-5p and erlotinib	9.87 ± 2.15*	6.54 ± 0.99*

IC_50_ values were determined by sigmoidal dose-response (variable slope) model using GraphPad Prism software. Data expressed as the mean±SD (n=3). *p<0.05 relative to the corresponding erlotinib group; #p>0.05 versus corresponding erlotinib.

## Discussion

Despite intensive advances in the treatment of lung cancer, it is remains an incurable disease. Owing to the occurrence of drug resistance in lung cancer cells, the survival rate still remains at low level (Mac Donagh et al., 2015; Wang et al., 2015). Therefore, development of new strategies for improved therapy is required. Overexpression of *EGFR* is attributed to the invasion, angiogenesis, proliferation, metastasis, and apoptosis resistance of many tumor cells including lung cancer (Yoshida et al., 2010; Seshacharyulu et al., 2012; Barr Kumarakulasinghe et al., 2015). Despite the therapeutic benefit of *EGFR* tyrosine kinase inhibitors, the efficacy of these agents is often limited by the development of drug resistance (Yoshida et al., 2010; Seshacharyulu et al., 2012; Antonicelli et al., 2013; Barr Kumarakulasinghe et al., 2015). However, the exact molecular mechanisms of resistance had remained unclear. In this study, we explored the effect of *miRNA-125a-5p* on *EGFR* expression, cell proliferation and sensitivity of NSCLC cells to erlotinib. 

qRT-PCR revealed that transfection of *miRNA-125a-5p *markedly reduced *EGFR mRNA* levels during the 3-day period. These data suggest that *miRNA-125a-5p* could effectively inhibit the expression of the EGFR, partly by decomposition of the corresponding mRNA. The results of the cell proliferation assay revealed that the up-regulation of *miRNA-125a-5p* significantly inhibited the proliferation of A549 cells, demonstrating its important role in the growth of lung cancer cells. Moreover, the results of MTT assay showed that pretreatment with *miRNA-125a-5p* distinctly decreased the IC_50_ value of erlotinib and subsequently enhanced its cytotoxicity. Combination study results clearly showed a synergistic interaction between *miRNA-125a-5p* and erlotinib at all concentrations of erlotinib.

To further explore the role of *miRNA-125a-5p* in the drug resistance of lung cancer cells, we examined the effect of* miRNA-125a-5p* on erlotinib-induced apoptosis. ELISA cell death assay revealed that erlotinib, alone, caused remarkable apoptosis in lung cancer cells. Of note, ELISA assay indicated that the inhibition of *EGFR *using *miRNA-125a-5p* also led to significant apoptosis in the absence of erlotinib. In addition, *miRNA-125a-5p*, in combination with erlotinib dramatically increased apoptosis level compared with *miRNA-125a-5p* alone or erlotinib alone. In contrast, neither NC miRNA nor lipofectamine changed the impact on drug sensitivity, which confirms the specific effect of *miRNA-125a-5p*. These data proposes that up-regulation of *miRNA-125a-5p *could sensitize the lung cancer cells to erlotinib via suppression of *EGFR*. 

Evidences suggests that dysregulation of miRNAs can be involved in the carcinogenesis and acquisition of resistance in cancer cells (MacDonagh et al., 2015). *MiRNA-125a *is a tumor suppressor that is transcripted from a gene on chromosome 19. Its tumor suppressive function was firstly confirmed in some types of cancers such as gastric, breast, glioblastoma and lung (Scott et al., 2007; Wang et al., 2009b; Cortez et al., 2010; Nishida et al., 2011; Wang et al., 2015). Previous studies showed that the expression of *miRNA-125a -3p*, one of the derivatives of *miRNA-125a*, was reduced in lung cancer cells and NSCLC tissues (Jiang et al., 2010a; LU et al., 2011). As to miRNA-125a-5p, another derivative of *miRNA-125a*, conflicting results have been observed. Jiang et al., (2010b) showed that the expression of *miRNA-125a-5p *was lower in lung cancer tissues than in adjacent normal tissues. The results of their study indicated that down-regulation of *miRNA-125a-5p* enhanced the migration and invasion of the lung tumor cells. Wang et al., (2009a) also found that *miRNA-125a-5p* is an *EGFR*-regulated *miRNA* that may function as a metastatic suppressor. The results of our study are in agreement with these reports and further confirm the negative correlation of *miRNA-125a-5p* with lung carcinogenesis. Nevertheless, conclusions from other studies which performed on the lung cancer cells are not consistent (Jiang et al., 2010b; LU et al., 2011).

The *EGFR* expression level has been shown to enhanced in many human malignancies (Yoshida et al., 2010; Barr Kumarakulasinghe et al., 2015). Some previous reports demonstrated that *miRNA-146a* and* miRNA-7* can inhibit the expression of *EGFR* and increase the sensitivity of the lung tumor cells to *EGFR* tyrosine kinase inhibitors (Rai et al., 2011; Chen et al., 2013). Another study showed that down-regulation of *miRNA-125a-5p* is associated with enhanced malignant potential such as tumor invasion, tumor size and poor prognosis in human gastric cancer (Nishida et al., 2011). However, other studies showed that down-regulation of *miRNA-125a-5p*, leads to an increase in the expression of *EGFR* and its downstream gene, enhancement of lung tumor cell migration and invasion (Wang et al., 2009b; Zhang et al., 2014; Wang et al., 2015). In this study, we showed that *miRNA-125a-5p* can inhibit the proliferation of the lung cancer cells and enhance the apoptotic effect of erlotinib by targeting *EGFR*. 

In conclusion, our study results indicate that *miRNA-125a-5p* inhibits *EGFR* expression in A549 cells, and that *miRNA-125a-5p* has the capacity to inhibit A549 growth in vitro. Down-regulation of *EGFR *by* miRNA-125a-5p* triggered significant apoptosis and enhanced sensitivity of the lung cancer cells to erlotinib in a synergistic way. Our data propose that the therapeutic delivery of *miRNA-125a-5p* may inhibit tumor proliferation, induce apoptosis and sensitize lung tumor cells to *EGFR* tyrosine kinase inhibitors.
